# Trends and risk factors of mortality analysis in patients with inflammatory bowel disease: a Taiwanese nationwide population-based study

**DOI:** 10.1186/s12967-019-02164-3

**Published:** 2019-12-12

**Authors:** Wei-Chen Lin, Meng-Tzu Weng, Chien-Chih Tung, Yuan-Ting Chang, Yew-Loong Leong, Yu-Ting Wang, Horng-Yuan Wang, Jau-Min Wong, Shu-Chen Wei

**Affiliations:** 1 Division of Gastroenterology, Department of Internal Medicine, MacKay Memorial Hospital, Taipei City, Taiwan; 2 MacKay Junior College of Medicine, Nursing and Management, Taipei, Taiwan; 3 MacKay Medical College, Taipei, Taiwan; 4 Department of Internal Medicine, National Taiwan University Hospital and College of Medicine, Taipei City, Taiwan; 5 School of Medicine, National Taiwan University, Taipei City, Taiwan; 6 Health Data Research Center, National Taiwan University, Taipei City, Taiwan; 7 Department of Internal Medicine, West Garden Hospital, Taipei City, Taiwan; 8 Inflammatory Bowel Disease Clinical and Study Integrated Center, National Taiwan University Hospital, Taipei City, Taiwan

**Keywords:** Crohn’s disease, Inflammatory bowel disease, Mortality, Risk factors, Ulcerative colitis

## Abstract

**Background:**

Inflammatory bowel disease (IBD) was emerging as a worldwide epidemic disease, and the advanced therapy changed the clinical course and possibly the outcomes. Our previous study reported a higher mortality rate from (IBD) in Taiwan than in Western countries. We proposed to analyze the trend and risk factors of mortality in order to improve the care quality of IBD patients.

**Methods:**

This retrospective study was conducted to analyze data for January 2001 to December 2015 from a registered database, compiled by the Taiwan’s National Health Insurance.

**Results:**

Between 2001 and 2015, a total of 3806 IBD patients [Crohn’s disease (CD): 919; ulcerative colitis (UC): 2887] were registered as having catastrophic illness, and 8.2% of these patients died during follow-up. The standardized mortality ratios (SMRs) of CD and UC were 3.72 (95% CI 3.02–4.55) and 1.44 (95% CI 1.26–1.65), respectively, from 2001 to 2015, respectively. A comparison of the periods of 2011–2015 and 2001–2005 revealed a decrease in the mortality rates from both UC and CD. Multivariate Cox proportional hazards analysis identified elderly individuals; sepsis and pneumonia were the risk factors for IBD mortality. The specific risk factors of mortality were liver cancer for UC and surgeries for CD.

**Conclusion:**

For further decreasing IBD-related mortality in Taiwan, we need to pay special attention toward elderly individuals, infection control, cancer screening and improvement in perioperative care.

## Introduction

Inflammatory bowel diseases (IBDs), which comprise ulcerative colitis (UC) and Crohn’s disease (CD), are chronic gastrointestinal disorders causing severe complications, such as toxic megacolon, sepsis, and thromboembolism [1]. Mortality from UC was sixfold higher than that from CD in 1951, and these rates have followed a parallel time course since 1975 [2]. Even with advancements in medical and surgical interventions, the mortality rates of IBD are still higher than what was previously understood [3]. A meta-analysis reported that the mortality rate from UC decreased and the survival rate was almost equal to that of the general population [4], whereas the mortality rate from CD remained high [5].

IBD can result in direct or indirect mortality. One-fifth of IBD-related are directly related to UC, which is less than a third of deaths caused by CD [6]. Mortality rates may vary geographically; for example, mortality rates are lower in Europe and North America because of the better healthcare system [6]. IBD mortality data from the Asia Pacific region are scant. Our previous study indicated a higher IBD mortality rate in Taiwan than in other Asian and Western countries, especially due to CD, from 1998 to 2008 [7]. For addressing this significant increase in the incidence and prevalence of IBD [7], we organized the IBD patient association to enhance patient knowledge of IBD symptoms, potential benefits and side effects of treatment since 2009. For physicians, we developed a series of comprehensive, practical education programs and on-line resources to improve the experience of gastroenterologists with management of IBD since 2010.

The primary aim of this study was to examine whether there has been a change in IBD mortality rate in Taiwan following the promotion of IBD-related educational programs since 2009. The secondary aim of the study was to analyze the risk factors of IBD deaths, which will help improve the quality of care for IBD patients.

## Methods

### Data source

Data were obtained from the Health and Welfare Data Science Center, Ministry of Health and Welfare (HWDC, MOHW). Established in 1995, the National Health Insurance (NHI) system in Taiwan is compulsory for all its residents and covers over 99% of the total population of approximately 23 million people. Since 1997, CD and UC are registered as catastrophic illnesses because of their potential for repetitive admissions and the need for chronic, careful care. Healthcare providers need to provide the patient’s clinical history record, endoscopic reports, image and histologic results when applying for the registration. NHI reviewers approve or decline the registration after reviewing the application. Therefore, not all clinical patients with IBD are registered, although most registered patients have confirmed IBD.

This nationwide population-based Taiwanese study of IBD was compiled between January 2001 and December 2015. NHI claims data provide clinical information including prescription, diagnoses, and hospitalizations for population-based epidemiologic research [8]. Under strict confidentiality guidelines and in accordance with personal electronic data protection regulations, the data of the study population were obtained from the HWDC, MOHW data (including Catastrophic Illness Registry and Taiwan Cancer Registry), Department of Statistics of the MOHW, Taiwan. This study was approved by the Institutional Review Board of the National Taiwan University Hospital (IRB Number 201507018 W). No informed consent is required under the Taiwan regulations for registry-based studies involving no contact with the study subjects.

## Patient identification

We used the diagnostic code [The International Classification of Diseases (ICD), Ninth and Tenth Revision, Clinical Modification] to retrieve data of the IBD patients (ICD-9 codes of 555 for CD and 556 for UC, other associated ICD codes are mentioned in Table 1) from the Catastrophic Illness Registry. For each patient, medical records were collected in the NHI claims database, including clinical diagnosis, as well as outpatient and hospitalization records. Age at IBD registration was defined as when patients were registered owing to catastrophic illnesses based on clinical, endoscopic, radiological, and histological features. Five most frequent comorbidities associated with mortality in Taiwan [hypertension, diabetes mellitus, hyperlipidemia, chronic obstructive pulmonary disease (COPD), and hepatitis] were included in the analysis. Extraintestinal manifestations (EIMs), namely uveitis, erythema nodosum, psoriasis, arthritis, cholangitis, deep venous thrombosis, and pulmonary embolism, were included in the analysis. We defined IBD complications as presence of fistula or abdominal abscess.

**Table 1:** Characteristics of IBD patients registered in the Catastrophic Illness Registry, Taiwan, 2001–2015

	ICD-9 and 10	Crohn’s disease	Ulcerative colitis	p value
		*N* (%)	*N* (%)	
Total		919 (100.0)	2887 (100.0)	
Gender				< 0.001
Male		631 (68.7)	1787 (61.9)	
Female		288 (31.3)	1100 (38.1)	
IBD registered age				< 0.001
≤ 39		543 (59.1)	1118 (38.7)	
40–59		241 (26.2)	1251 (43.3)	
≥ 60		135 (14.7)	518 (17.9)	
Operation				
Colectomy	73014B	6 (0.6)	9 (0.3)	0.221
Colostomy	73022B	73 (7.9)	122 (4.2)	< 0.001
Exploratory laparotomy	75805B	25 (2.7)	13 (0.5)	< 0.001
Ileostomy	73,015-7B	27 (2.9)	78 (2.7)	0.703
Comorbidity				
Hypertension	401; A260, 269; I10	166 (18.1)	674 (23.3)	0.001
Diabetes	250; A181;E101-19,131	87 (9.5)	326 (11.3)	0.121
Hyperlipidemia	272, A189;E752,77-8,88	121 (13.2)	599 (20.7)	< 0.001
COPD	491-2; A323;J41-4	58 (6.3)	229 (7.9)	0.105
Hepatitis	70; A46; B15-9	62 (6.7)	251 (8.7)	0.061
EIM				
Uveitis	360; A239; H440-9	23 (2.5)	90 (3.1)	0.339
Psoriasis	696; A429; L305,40-8	56 (6.1)	136 (4.7)	0.095
Erythema nodosum	695.2; A429; L52	25 (2.7)	22 (0.8)	< 0.001
Arthritis	714; A430; M500-840,1200	84 (9.1)	193 (6.7)	0.013
Cholangitis	576.1; K830	15 (1.6)	27 (0.9)	0.078
Deep vein thrombosis	453, 451.1, A303, I820-9	16 (1.7)	32 (1.1)	0.135
Pulmonary embolism	415.1; T800; I2690-9	3 (0.3)	6 (0.2)	0.519
Complication				
Fistula	569.8, 596.1, 565, K602-5 632,	258 (28.1)	381 (13.2)	< 0.001
Abdominal wall abscess	566; 569.5; K610-3630	121 (13.2)	107 (3.7)	< 0.001
Cancer				
Small intestine cancer	152	0 (0.0)	0 (0.0)	^b^
Colorectal cancer	153; 154	8 (0.8)	21 (0.7)	0.664
Liver cancer	155	16^a^		0.275
Lymphoma	200; 202	4 (0.4)	11 (0.4)	0.767
Melanoma	172.9	0 (0.0)	0 (0.0)	^b^
Hospitalization				
Pneumonia	482-6; A321, 481;J14-8	92 (10.0)	210 (7.3)	0.008
Urinary tract infection	599; A359; N390	111 (12.1)	243 (8.4)	< 0.001
Sepsis	038; A038, 409-19	169 (18.4)	293 (10.2)	< 0.001
Survival state				0.003
Survival		823 (89.5)	2673 (92.6)	
Mortality		96 (10.5)	214 (7.4)	

^a^One group was less than 3 patients and it was not allowed to mention it in detail by the regulations of the Health and Welfare Data Science Center, ^b^data not applicable

## Statistical analyses

We described the characteristics of IBD patients, including the year of IBD registered, sex ratio, and age distribution, in this study. Mortality was compared with that in the survival group by using the independent *t* test and Chi square test. The Cox proportional hazards model was applied to analyze the effect of multiple covariates in predicting mortality in patients with IBD. The adjusted hazard ratio was dependent on each factor of the significant univariate factors in UC and CD patients. The survival of IBD patients was assessed using the Kaplan–Meier life-table method, and the differences were evaluated using the log rank test. To evaluate the trends of mortality rate and the effect of age on mortality, the study was divided into 3 consecutive periods of 5 years (2001–2005, 2006–2010, and 2011–2015) and 3 age groups (≤ 39, 40–59,  ≥ 60 years). The mortality rate was defined as the number of IBD deaths per 1000 persons per year. Specific mortality rates were calculated as the death rates of the specific group among patients. Standardized mortality ratios (SMRs) were estimated as the observed number of deaths divided by the expected number of deaths, and 95% confidence intervals (CIs) were calculated using Poisson distribution. The standard population was the national population, and the number of expected deaths was calculated on the basis of the number of deaths and number of people in the middle of the year in Taiwan. All statistical analyses were performed using SAS version 9.4 (SAS Institute, Cary, NC, USA). A p value of < 0.05 was considered statistically significant.

## Results

### Clinical characteristics

Between 2001 and 2015, a total of 3806 newly diagnosed IBD patients were registered, and their demographic data are summarized in Table 1. Of the total patients, 919 were diagnosed as having CD and 2887 as having UC. The patients were predominantly men, with CD being significantly more prevalent than UC (p < 0.001). The male-to-female ratio for CD and UC was 2.2 and 1.6, respectively (Table 1). The age at disease onset or registration was higher in UC patients than in CD patients (p < 0.001). The incidence rate of CD and UC peaked at the ages of ≤ 39 and 40–59 years, respectively (Table 1).

UC patients had more comorbidities (hypertension and hyperlipidemia) than CD patients. Erythema nodosum and arthritis were the more common EIMs in CD patients. No significant difference was observed with respect to malignancy between the UC and CD groups. Colostomy and explorative laparotomy as surgeries; fistula and abdominal wall abscess as complications; and infection (pneumonia, urinary tract infection, and sepsis)-related hospitalizations occurred more frequently in the CD group (Table 1), resulting in CD patients having a higher mortality rate than UC patients (10.5% vs. 7.4%; p = 0.003).

### Univariate analysis of risk factors for mortality

Of the 310 patients (8.2%) who died during follow-up, 96 had CD and 214 had UC (Table 2). Women had excess mortality in CD when the survival and mortality groups were compared (p = 0.038). Because 61.7% UC and 52.1% CD patients died older than 60 years (Table 2), age was deemed to be a significant factor affecting mortality (p < 0.001 and p < 0.001). The mortality group tended to have received colostomy, exploratory laparotomy, and ileostomy. The rates of comorbidities such as hypertension and diabetes; and infections such as sepsis, pneumonia, and urinary tract infection occurred more frequently in the mortality group. In EIM, cholangitis in UC and deep venous thrombosis in CD were the risk factors of mortality (p = 0.003 and p = 0.019). Deep vein thrombosis and pulmonary embolism (PE) in UC were higher in the mortality group but didn’t reach statistical difference. Malignancies such as colorectal cancer, liver cancer and lymphoma were related to the IBD mortality with statistical difference, especially in UC patients. Due to the small cases of lymphoma and pulmonary embolism, the detail was not presented in the Table 2. Surprisingly, fistula as a disease-related complication seemed to occur more frequently in the survival group of CD (p < 0.001). The mortality group more frequently experienced malignancy.

**Table 2:** Risk factors of mortality for IBD patients registered in the Catastrophic Illness Registry, Taiwan, 2001–2015

	UC (*n* = 2887)			CD (*n* = 919)		
	Mortality (*n *= 214)	Survival (*n *= 2673)	p value	Mortality (*n *= 96)	Survival (*n *= 823)	p value
	*n* (%)	*n* (%)		n (%)	n (%)	
Male sex	143 (66.8)	1644 (61.5)		57 (59.4)	574 (69.7)	0.038
IBD registered age			< 0.001			< 0.001
≤ 39	24 (11.2)	1094 (40.9)		20 (20.8)	523 (63.6)	
40–59	58 (27.1)	1193 (44.6)		26 (27.1)	215 (26.1)	
≥ 60	132 (61.7)	386 (14.4)		50 (52.1)	85 (10.3)	
Operation						
Colectomy	1 (0.5)	8 (0.3)	0.501	1 (1.0)	5 (0.6)	0.485
Colostomy	31 (14.5)	91 (3.4)	< 0.001	21 (21.9)	52 (6.3)	< 0.001
Exploratory laparotomy	4 (1.9)	9 (0.3)	0.012	9 (9.4)	16 (1.9)	< 0.001
Ileostomy	17 (7.9)	61 (2.3)	< 0.001	8 (8.3)	19 (2.3)	0.004
Comorbidity						
Hypertension	94 (43.9)	580 (21.7)	< 0.001	37 (38.5)	129 (15.7)	< 0.001
Diabetes	48 (22.4)	278 (10.4)	< 0.001	19 (19.8)	68 (8.3)	< 0.001
Hyperlipidemia	42 (19.6)	557 (20.8)	0.674	20 (20.8)	101 (12.3)	0.019
COPD	41 (19.2)	188 (7.0)	< 0.001	10 (10.4)	48 (5.8)	0.081
Hepatitis	19 (8.9)	232 (8.7)	0.921	7 (7.3)	55 (6.7)	0.829
EIM						
Uveitis	0 (0.0)	90 (3.4)	0.006	0 (0.0)	23 (2.8)	0.159
Psoriasis	10 (4.7)	126 (4.7)	0.978	2 (2.1)	54 (6.6)	0.111
Erythema nodosum	2 (0.9)	20 (0.8)	0.763	1 (1.0)	24 (2.9)	0.504
Arthritis	21 (9.8)	172 (6.4)	0.057	7 (7.3)	77 (9.4)	0.507
Cholangitis	7 (3.3)	20 (0.8)	0.003	4 (4.2)	11 (1.3)	0.062
Deep vein thrombosis	5 (2.3)	27 (1.0)	0.083	5 (5.2)	11 (1.3)	0.019
Complication						
Fistula	26 (12.2)	355 (13.3)	0.638	13 (13.5)	245 (29.8)	< 0.001
Abdominal wall abscess	11 (5.1)	96 (3.6)	0.249	7 (7.3)	114 (13.9)	0.072
Cancer						
Colorectal cancer	10 (4.7)	11 (0.4)	< 0.001	3 (3.1)	5 (0.6)	0.042
Liver cancer	9 (4.2)	5 (0.2)	< 0.001	2 (2.1)	0 (0.0)	0.011
Hospitalization						
Pneumonia	96 (44.9)	114 (4.3)	< 0.001	36 (37.5)	56 (6.8)	< 0.001
Urinary tract infection	69 (32.2)	174 (6.5)	< 0.001	25 (26.0)	86 (10.5)	< 0.001
Sepsis	122 (57.0)	171 (6.4)	< 0.001	57 (59.4)	112 (13.6)	< 0.001

### Multivariate analysis of risk factors for mortality

In the multivariate analysis, pulmonary embolism became a risk factor of mortality in UC. After adjusting the significant univariate factors, the multivariable logistic regression model indicated that mortality in IBD was associated with age, lymphoma, pneumonia and sepsis (Table 3). Among these covariates, the hazard ratio (HR) of mortality in CD increased significantly with lymphoma (HR: 12.26; 95% CI 2.85–52.63), followed by age groups ≥ 60 years (HR: 11.93; 95% CI 6.25–22.79) and sepsis (HR: 5.36; 95% CI 3.41–8.41). In UC patients, the mortality increased significantly with age groups ≥ 60 years (HR: 8.93; 95% CI 5.50–14.52), followed by sepsis (HR: 5.40; 95% CI 3.86–7.56) and pulmonary embolism (HR: 4.21; 95% CI 1.01–17.57). The operation of colostomy, ileostomy and exploratory laparotomy was still the significant risk factor of mortality in CD, while liver cancer was related to mortality in UC. There was no significant difference in the risk factors of cholangitis, deep venous thrombosis, comorbidity and colorectal cancer.

**Table 3:** Multivariable hazard ratios of mortality for IBD patients registered in the Catastrophic Illness Registry, Taiwan, 2001–2015

	UC				CD			
	cHR	95% CI	aHR^a^	95% CI	cHR	95% CI	aHR^b^	95% CI
Gender								
Female	1.00	(Ref.)			1.00	(Ref.)	1.00	(Ref.)
Male	1.29	(0.97–1.71)			0.65	(0.43–0.97)	1.04	(0.65–1.66)
IBD registered age								
≤ 39	1.00	(Ref.)	1.00	(Ref.)	1.00	(Ref.)	1.00	(Ref.)
40–59	2.23	(1.39–3.59)	2.21	(1.37–3.59)	2.91	(1.63–5.22)	3.11	(1.69–5.72)
≥ 60	14.16	(9.16–21.88)	8.93	(5.50–14.52)	13.95	(8.28–23.50)	11.93	(6.25–22.79)
Operation								
Colostomy	3.67	(2.51–5.37)	1.28	(0.83–1.95)	3.66	(2.26–5.95)	2.27	(1.33–3.87)
Exploratory laparotomy	5.04	(1.87–13.59)	2.41	(0.84–6.88)	3.57	(1.79–7.10)	2.27	(1.04–4.98)
Ileostomy	2.69	(1.64–4.42)	1.34	(0.79–2.27)	3.13	(1.51–6.45)	2.26	(1.04–4.90)
Comorbidity								
Hypertension	2.35	(1.80–3.08)	0.69	(0.50–0.95)	2.96	(1.96–4.47)	0.68	(0.39–1.19)
Diabetes	2.17	(1.57–2.99)	0.76	(0.53–1.09)	2.33	(1.41–3.85)	0.67	(0.35–1.28)
Hyperlipidemia	0.84	(0.60–1.18)			1.66	(1.01–2.71)	0.76	(0.41–1.40)
COPD	2.52	(1.79–3.55)	0.82	(0.56–1.18)	1.70	(0.88–3.28)		
EIM								
Cholangitis	3.81	(1.79–8.09)	2.14	(0.95–4.81)	2.35	(0.86–6.40)		
Deep vein thrombosis	1.99	(0.82–4.83)			3.14	(1.28–7.74)	1.14	(0.44–2.98)
Pulmonary embolism	8.76	(2.17–35.43)	4.21	(1.01–17.57)	4.75	(0.66–34.24)		
Complication								
Fistula	0.91	(0.60–1.37)			0.38	(0.21–0.68)	0.60	(0.32–1.10)
Cancer								
Colorectal cancer	6.61	(3.50–12.49)	1.84	(0.94–3.61)	3.46	(1.09–10.92)	1.04	(0.27–3.96)
Liver cancer	8.59	(4.40–16.75)	2.16	(1.05–4.42)	14.96	(3.67–60.95)	1.80	(0.36–9.07)
Lymphoma	9.25	(3.81–22.48)	3.65	(1.45–9.19)	7.38	(1.81–30.03)	12.26	(2.85–52.63)
Hospitalization								
Pneumonia	11.23	(8.57–14.70)	2.88	(2.08–3.99)	5.27	(3.48–7.98)	1.79	(1.13–2.85)
Urinary tract infection	5.18	(3.89–6.91)	0.87	(0.62–1.22)	2.52	(1.60–3.97)	0.78	(0.44–1.38)
Spesis	13.09	(9.98–17.16)	5.40	(3.86–7.56)	6.53	(4.34–9.81)	5.36	(3.41–8.41)

*cHR* crude hazard ratio, *aHR* adjusted hazard ratio, *CI* confidence interval

^a^Adjusted risk factor: IBD registered age, Colostomy, Exploratory laparotomy, Ileostomy, Hypertension, Diabetes, COPD, Cholangitis, Pulmonary embolism, Colorectal cancer, Liver cancer, lymphoma, Pneumonia, Urinary tract infection, Sepsis

^b^Adjusted risk factor: Gender, IBD registered age, Colostomy, Exploratory laparotomy, Ileostomy, Hypertension, Diabetes, Hyperlipidemia, Deep vein thrombosis, Fistula, Colorectal cancer, Liver cancer, lymphoma, Pneumonia, Urinary tract infection, Sepsis

### Mortality rate between different time periods and groups

**Fig. 1:**
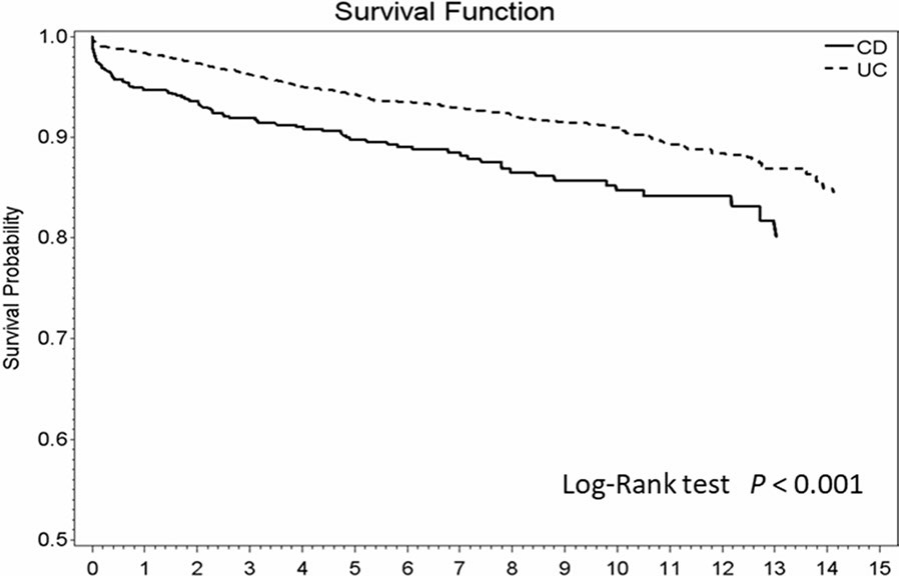
Survival rate of patients registered in the Catastrophic Illness Registry with IBD, Taiwan, 2001–2015

CD and UC death rates decreased with time on categorizing mortality into 3 time periods (2001–2005, 2006–2010, and 2011–2015; Table 4). The mortality rate of CD decreased from 21.2/1000 person-years to 19.9/1000 person-years during 2001–2005 and 2011–2015 (p = 0.002). The mortality rate of UC was 11.6/1000 persons-years during 2001–2005 and 8.5/1000 persons-years during 2011–2015 (p = 0.020). The mortality rate during 2006–2010, prior to the introduction of the training/online resources in 2010, appeared to decrease slightly in UC and CD, but the difference in the SMR was not statistically significant between the periods 2001–2005 and 2006–2010. The survival curve of CD and UC patients during this study period is shown in Fig. 1. The 5-, 10-, and 13-year survival rates of CD patients after registration were 90%, 85%, and 80%, respectively, whereas those of UC patients were 95%, 91%, and 87%, respectively. The survival rate was significantly higher in the UC group than in the CD group from 2001 to 2015 (p < 0.001).

**Table 4:** Mortality in IBD patients registered in Catastrophic Illness Registry between 2001 and 2015, Taiwan

Dx year	Crohn’s disease					Ulcerative colitis				
	Dx (*n*)	Death (*n*)	Person-year	Mortality rate (per 1000 person-year)	p value	Dx (*n*)	Death (*n*)	Person-year	Mortality rate (per 1000 person-year)	p value
2001–2005	205	45	2125	21.2	0.002^a^	886	117	10,066	11.6	0.020^a^
2006–2010	258	32	1714	18.7		1077	79	7572	10.4	
2011–2015	456	19	955	19.9		924	18	2110	8.5	

^a^Comparison disease year of 2001–2005 and 2011–2015

**Fig. 2:**
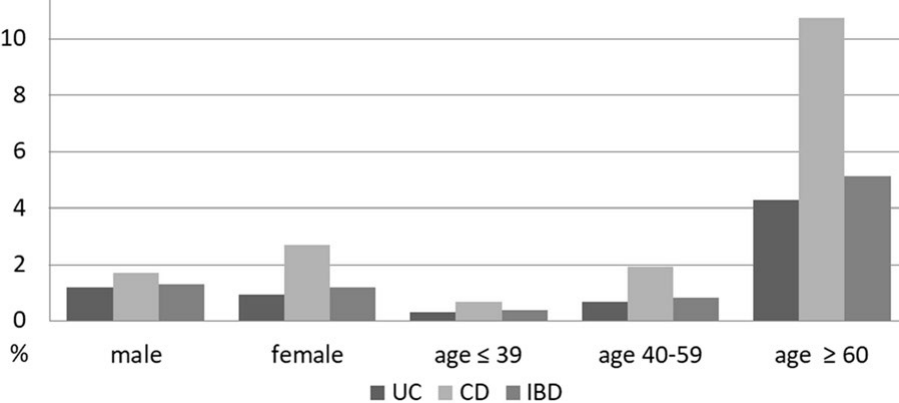
Age- and gender-stratified specific mortality rate of patients registered in the Catastrophic Illness Registry with IBD, Taiwan, 2001–2015

In the age- and gender-stratified specific mortality analysis (Fig. 2), gender did not affect the mortality rate, although men had a higher mortality rate in the UC group (1.19% vs. 0.92%) and women had a higher mortality rate in the CD group (1.70% vs. 2.70%). When stratified for age, the mortality rates of both UC and CD groups increased with age, with ≥ 60-year-old CD patients having the highest mortality rate of 10.74%.

### SMRs of IBD patients

**Fig. 3:**
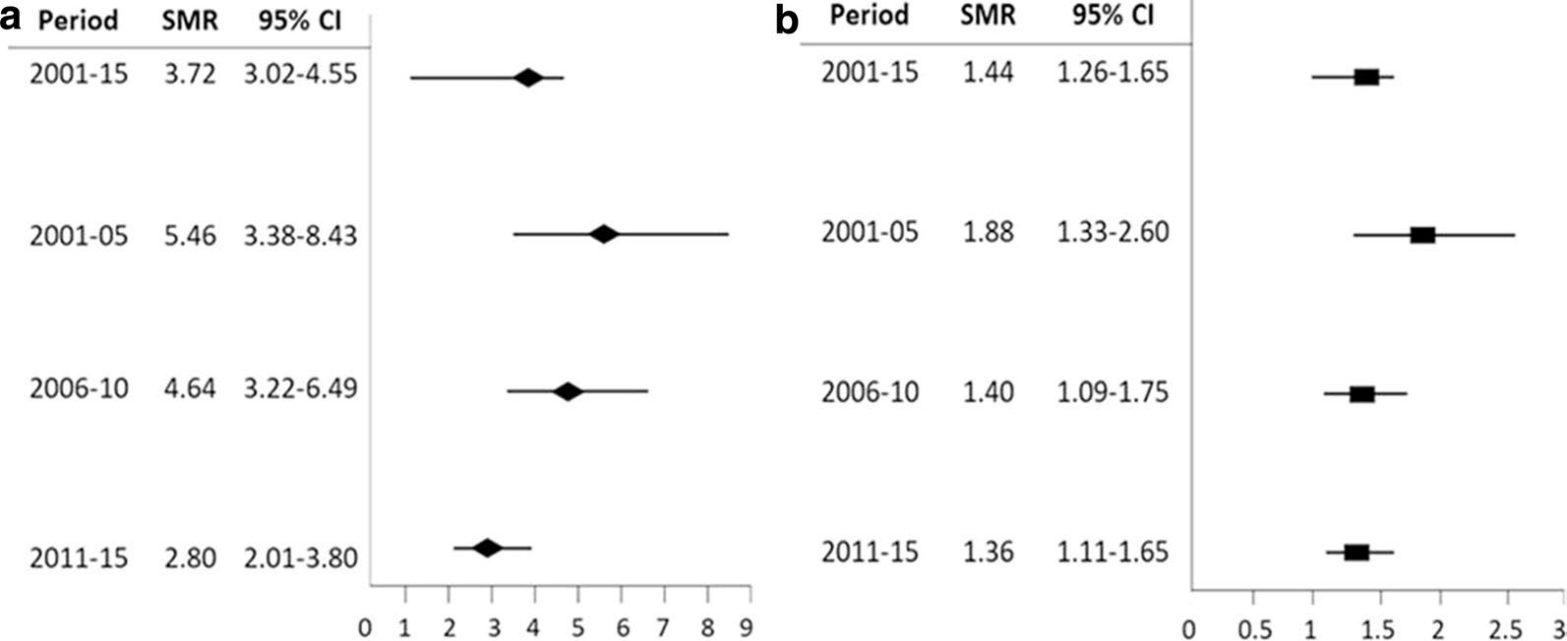
Standard mortality ratio of **a** Crohn’s disease and **b** ulcerative colitis in different time periods

The SMRs for all-cause mortality in CD and UC patients during 2001–2015 were 3.72 (95% CI 3.02–4.55) and 1.44 (95% CI 1.26–1.65), respectively (Fig. 3). When stratified for different time periods, the SMRs of CD and UC decreased from 5.46 to 2.80 and from 1.88 to 1.36, respectively, during 2001–2005 and 2011–2015.

## Discussion

Our nationwide cohort study demonstrated a significant decrease in CD and UC mortality rates in Taiwan during 2001 to 2015. The mortality rate was however higher in CD patients. The CD group had higher infection and surgery rates, whereas the UC group had more comorbidities. Older age, sepsis and pneumonia were the risk factors for mortality in the multivariate analysis. Surgeries were the significant risk factors in CD, while liver cancer was a risk factor in UC. For decreasing the mortality rates associated with IBD, it is vital to recognize and treat the early clinical warning signs of the disease. Our study will assist clinicians in understanding better the risk factors of mortality in IBD patients.

The disease severity of IBD in Asia seems to be equal to or greater than that in the West [9]. Our study reported a higher mortality rate in CD patients, in accordance with findings from previous studies [10, 11]. With the increase in IBD incidence over the years in Taiwan, physician awareness of the disease has increased, especially through continuous medical education programs for IBD since 2009. A previous study reported an increasing trend in the use of immunosuppressant agents for treating UC over the years in Taiwan: 0% (1998) to 7% (2008) [12]. A population-based study revealed that immunosuppressant therapy could significantly improve IBD survival rates [13]. Biologics have been demonstrated to aid mucosal healing and decrease the incidence of IBD-related complications following surgery [14]. Adalimumab was the first biologic to be reimbursed by the NHI since 2011 for treating CD, followed by more biologics for the treatment of UC and CD since 2016 [15]. Because of budget limitations, the NHI allows the use of biologics only for a limited period; however, in case of clinical relapse, the patient can apply again [15]. A recent study from Taiwan showed that a high accumulated dose of anti-TNF-α agents and corticosteroid is associated with increased risk of operation [16], which may be because of the severe disease activity and late use of biologics.

A previous study showed that infection-associated mortality was higher in patients with CD than in those with UC [10]. Sepsis and pneumonia were the most common causes of infection-related mortality and abdominal abscesses did not seem to increase the mortality rate [17]; as observed in our study. It was well recognized the incidence of thromboembolic event (VTE) in IBD patients was lower in Asia than in Western countries [18]. Routine thromboprophylaxis was not a standard practice in Asia, while our study showed pulmonary embolism (PE) played an important risk factor of mortality for UC. Therefore, we suggested to closely monitor the possibility of VTE in the admitted IBD patients. In selective condition, for example, when with more risk factors of PE (surgery, malignancy, hormone change- for example, pregnancy or hormone replacement, thrombophilia, and immobilization) [19], prophylaxis might be indicated. In this study, a higher rate of comorbidity in the UC group may be attributed to the higher mean age of UC patients than that of CD patients. A population-based Swiss study revealed a high prevalence of concomitant chronic diseases in IBD patients compared to non-IBD patients; 78% of IBD patients had at least one comorbidity, with a median of three comorbidities [20].

In our study, ostomy or exploratory laparotomy was a risk factor for mortality in CD patients. This result may reflect the fact that this surgery was performed in an emergency or for refractory IBD. Ostomy construction is an effective treatment modality for refractory large bowel CD or perianal CD [21]. A systematic review reported that emergent surgery for IBD is associated with higher mortality rates [22]. Postoperative mortality decreased significantly over time for CD patients, but not for UC patients [22, 23]. Optimization of medical treatment, especially perioperative care, is necessary for decreasing ostomy-related mortality.

Fistula is a severe complication of CD, and it is associated with increased morbidity and impairment in health-related quality of life [24]. In this study, it appeared that fistula was not a factor of mortality. A literature-based analysis showed that anal fistulas were the most common type of fistula [25]. Simple anal fistulas are associated with a better prognosis and are less likely to require radical surgical procedures than complex fistulas [26]. Perianal disease is more commonly detected in East Asian countries than in Western countries [27]. Since patients with perianal disease might seek and adhere to treatment, this might explain why the presence of fistula did not cause mortality in this study.

Long-standing IBD has an increased risk of developing colorectal cancer (CRC) and CRC is associated with increased mortality [28, 29]. Other cancers, such as lung cancer, biliary tract tumor, and lymphoma, are also related to the mortality on IBD [11, 28, 29]. Colon cancer, lung cancer, and liver tumor are reported to be the top five common cancers in Taiwan, whereas lymphoma shows an increasing trend of incidence [30]. In this study, we found that liver cancer plays a central role in UC mortality, while CRC was not related to mortality in the multivariate analysis. We searched for liver cancer by using the code 155, which included hepatocellular carcinoma and cholangiocarcinoma. Primary sclerosing cholangitis in IBD patients was a well-known risk factor of cholangiocarcinoma, especially in those with UC. Higher cholangitis rate was also observed in mortality group in UC patients. It is reasonable for liver cancer presented with a high hazard ratio of mortality in UC patients. We also observed CD patients had higher hazard ratios of surgery than UC patients, which suggested that CD patients had more severe disease activity and were more likely to receive immunosuppressants and anti-TNF agents that increased their risk of lymphoma. Due to the reimbursement criteria, more CD patients received thiopurine and anti-TNF agents than UC patients in Taiwan during this study period [16]. We did not identify any melanoma or non-melanoma skin cancer in our IBD patients during follow-up because of the rarity of malignant melanoma in Asia. Regarding extraintestinal cancers, surveillance programs for malignancy are needed to closely monitor IBD patients.

One review article that included studies conducted during 1980–2010 demonstrated an SMR of approximately 1.5-fold (range, 0.7–2.2) for CD patients, whereas the SMR for UC patients was similar to that of the general population (range, 0.7–1.7) [6]. IBD mortality and treatment outcomes might have changed with the development of biologics since 1998. In Taiwan, the SMRs for CD and UC were 3.72 and 1.44, respectively, higher than that reported in Korea and other Western countries during the same period [28, 29, 31, 32]. With the introduction of training/online resources in 2010 and the introduction of biologics reimbursement in Taiwan in 2011, the SMRs of CD and UC gradually decreased to 2.80 and 1.36, respectively. Realizing the risk factors of mortality and proper management of complications are important to lower IBD-related mortality, especially in CD.

The strength of this study was the availability of a large, nationwide cohort of individuals with IBD from a country that provides access to healthcare and registration for all its citizens. However, there are limitations in our study. First, elderly people tend to have more comorbidities; therefore, aging itself as the exact cause of death may oversimplify the reality. The exact cause of death is difficult to determine and complex; therefore, our study chose the contributing risk factors of death. Second, only IBD patients who had a certificate for catastrophic illnesses were registered, and those with mild illnesses were excluded. This may have led to an overestimated mortality rate and delayed age of disease onset (registered). Third, detailed clinical data such as phenotype, laboratory results, disease duration, and smoking and family histories were not available for the included patients. Fourth, IBD follow-up periods for biologics use, comorbidities, and malignancies were short, which impeded the analysis of natural course patterns. Nevertheless, our results could be useful in countries with an increasing incidence and prevalence of IBD where physicians are beginning to gain insights into IBD treatment. Education on IBD, as in the pre-biologic era, can help decrease disease-related mortality.

## Conclusion

This is the first Taiwanese nationwide database study focusing on the risk factors of IBD mortality. Although CD patients had higher mortality, the mortality rates of both CD and UC were significantly decreased from 2001 to 2015. Awareness of the disease in elderly individuals, infection control, and improvement in perioperative care could help decrease IBD-related mortality.

## Data Availability

The datasets generated during and/or analysed during the current study are available in the Health and Welfare Data Science Center, Ministry of Health and Welfare repository, Taiwan. (https://dep.mohw.gov.tw/DOS/lp-2506-113.html).

## Abbreviations

Anti-TNF-α: anti-tumor necrosis factor alpha
CD: crohn’s disease
CI: confidence interval
COPD: chronic obstructive pulmonary disease
CRC: colorectal cancer
EIMs: extraintestinal manifestations
HR: hazard ratio
HWDC: Health and Welfare Data Science Center
IBD: inflammatory bowel disease
ICD: international classification of diseases
MOHW: Ministry of Health and Welfare
NHI: National Health Insurance
PE: pulmonary embolism
SMRs: standardized mortality ratios
UC: ulcerative colitis
VTE: venous thromboembolic event
